# Supplementary material for: Enhancing Prognosis in Advanced Ovarian Cancer: Primary Cytoreductive Surgery and Adjuvant Chemotherapy or Neoadjuvant Chemotherapy and Interval Cytoreduction—A Single-Center Retrospective Observational Study
Source: Cancers (Basel). 2025 Apr 14;17(8):1314. doi: 10.3390/cancers17081314 (PMC12026333; doi:10.3390/cancers17081314)
Supplement: Supplementary file 1 [file cancers-17-01314-s001.zip › cancers-3507646-supplementary.pdf]

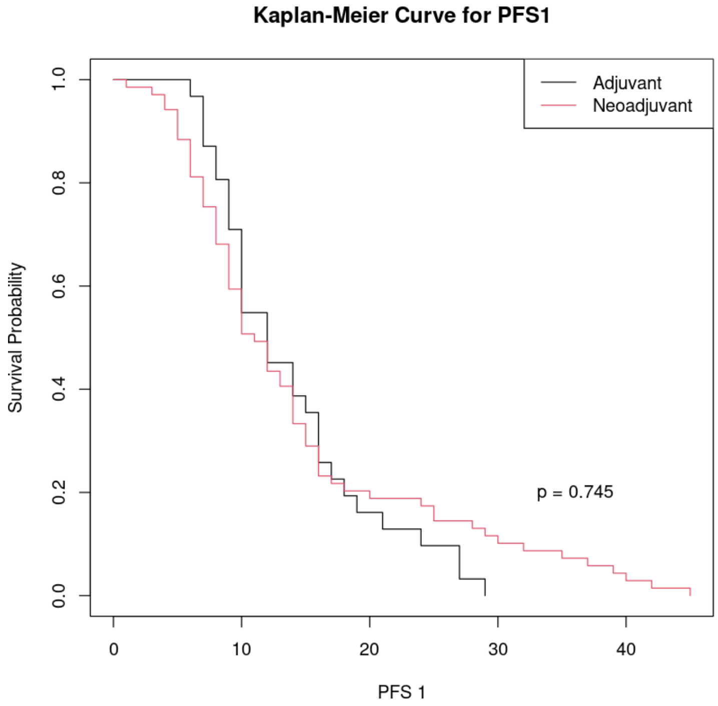

Figure S1. Kaplan-Meier curve comparing progression-free survival 1 (PFS1).

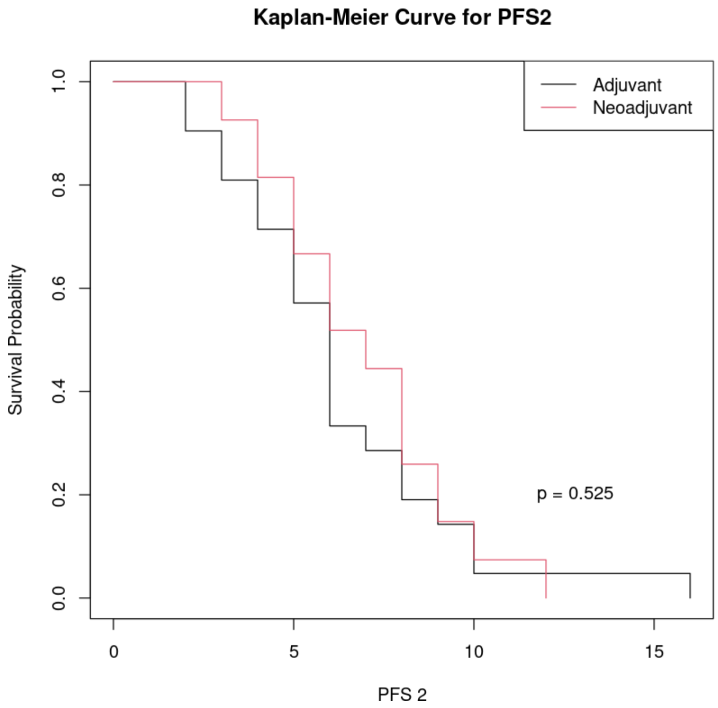

Figure S2. Kaplan-Meier curve comparing progression-free survival 2 (PFS2).

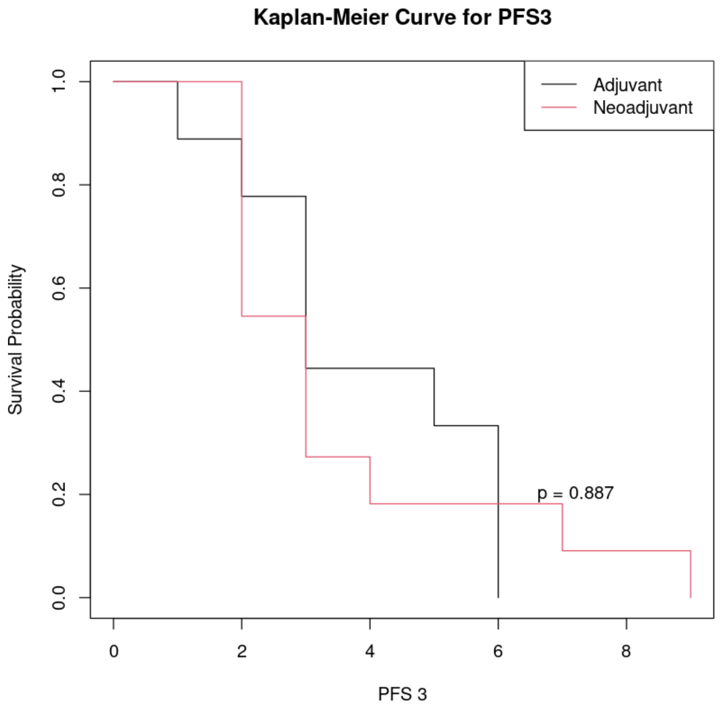

Figure S3. Kaplan-Meier curve comparing progression-free survival 3 (PFS3).

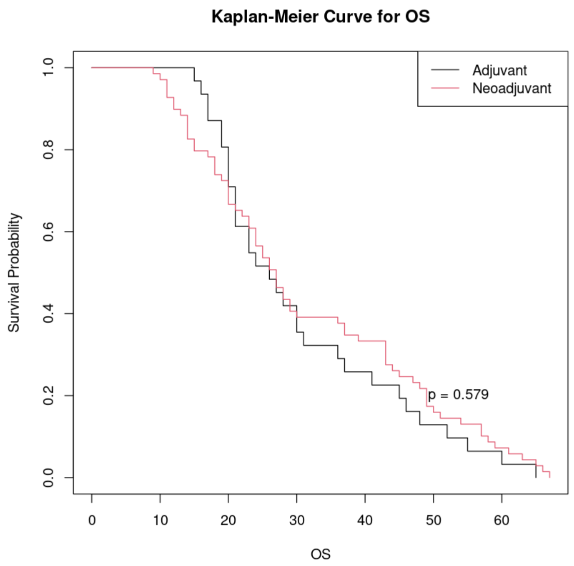

Figure S4. Kaplan-Meier curve comparing overall survival (OS).

Table S1. Influence of COVID-19 on ovarian cancer patients.

	n	% (n/N*100%)	95% CI inf	95% CI sup

The influence of COVID-19				
No influence	87	87,00%	78,80%	92,89%
Treatment temporization	7	7,00%	2,86%	13,89%
Deaths	6	6,00%	2,23%	12,60%
	**N=100**	**100,00%**		

95%. CI inf = lower limit of the 95% confidence interval.

95%. CI sup = upper limit of the 95% confidence interval.

Table S2. Perceived risks, fears and opinions about the influence of COVID-19 on cancer progression.

	**n**	**% (n/N*100%)**	**95% CI inf**	**95% CI sup**
**Do you consider that your cancer diagnosis puts you at an extra risk compared to the rest of the population for the new coronavirus?**				
Yes	54	67,50%	56,11%	77,55%
No	12	15,00%	8,00%	24,74%
I don't know	14	17,50%	9,91%	27,62%
	N = 80	100.00%		
**Do you consider that the risk of getting infected with coronavirus is a reason to delay/withhold cancer treatment until the pandemic is over?**				
Yes	3	3,80%	0,79%	10,70%
No	70	88,61%	79,47%	94,66%
I don't know	6	7,59%	2,84%	15,80%
	N = 79	100.00%		
**What are you most afraid of?**				
The progression of cancer disease	28	35,00%	24,67%	46,48%
Coronavirus infection	6	7,50%	2,80%	15,61%
Both in equal measure	46	57,50%	45,94%	68,49%
	N = 80	100.00%		

95%. CI inf = lower limit of the 95% confidence interval.

95%. CI sup = upper limit of the 95% confidence interval.
